# Ovine Mesenchymal Stromal Cells: Morphologic, Phenotypic and Functional Characterization for Osteochondral Tissue Engineering

**DOI:** 10.1371/journal.pone.0171231

**Published:** 2017-01-31

**Authors:** Clara Sanjurjo-Rodríguez, Rocío Castro-Viñuelas, Tamara Hermida-Gómez, Tania Fernández-Vázquez, Isaac Manuel Fuentes-Boquete, Francisco Javier de Toro-Santos, Silvia María Díaz-Prado, Francisco Javier Blanco-García

**Affiliations:** 1 Cell Therapy and Regenerative Medicine Unit, Rheumatology Group, Institute of Biomedical Research of A Coruña (INIBIC), University Hospital Complex A Coruña (CHUAC), Galician Health Service (SERGAS), Department of Medicine, Faculty of Health Sciences, University of A Coruña, A Coruña, Spain; 2 Tissue Bioengineering and Cell Therapy Unit (GBTTC-CHUAC), Rheumatology Group, Institute of Biomedical Research of A Coruña (INIBIC), University Hospital Complex A Coruña (CHUAC), Galician Health Service (SERGAS), A Coruña, Spain; University of Connecticut Health Center, UNITED STATES

## Abstract

**Introduction:**

Knowledge of ovine mesenchymal stromal cells (oMSCs) is currently expanding. Tissue engineering combining scaffolding with oMSCs provides promising therapies for the treatment of osteochondral diseases.

**Purpose:**

The aim was to isolate and characterize oMSCs from bone marrow aspirates (oBMSCs) and to assess their usefulness for osteochondral repair using β-tricalcium phosphate (bTCP) and type I collagen (Col I) scaffolds.

**Methods:**

Cells isolated from ovine bone marrow were characterized morphologically, phenotypically, and functionally. oBMSCs were cultured with osteogenic medium on bTCP and Col I scaffolds. The resulting constructs were evaluated by histology, immunohistochemistry and electron microscopy studies. Furthermore, oBMSCs were cultured on Col I scaffolds to develop an *in vitro* cartilage repair model that was assessed using a modified International Cartilage Research Society (ICRS) II scale.

**Results:**

oBMSCs presented morphology, surface marker pattern and multipotent capacities similar to those of human BMSCs. oBMSCs seeded on Col I gave rise to osteogenic neotissue. Assessment by the modified ICRS II scale revealed that fibrocartilage/hyaline cartilage was obtained in the *in vitro* repair model.

**Conclusions:**

The isolated ovine cells were demonstrated to be oBMSCs. oBMSCs cultured on Col I sponges successfully synthesized osteochondral tissue. The data suggest that oBMSCs have potential for use in preclinical models prior to human clinical studies.

## Introduction

Articular cartilage and its supporting bone are tightly coupled, forming a connected osteochondral unit [1]. Orthopaedic surgeons have recently focused on the treatment of osteochondral lesions because most of these lesions do not heal spontaneously and can develop into osteoarthritis [1, 2]. Several treatment approaches have been tested, including osteochondral autologous transplantation or microfracture [3, 4]. However, none of the currently available approaches have achieved clinical acceptance for repair of the osteochondral unit [1, 5, 6]. This lack of effective treatment motivates research into the tissue engineering of a biological implant to replace the diseased joint [7, 8]. In tissue engineering, scaffolds are indispensable as carriers of cells at the injured site that stimulate neotissue formation [9]. In addition, scaffolds provide a comfortable niche for cells, stimulating them to synthesize matrix and replace the function of the native tissue [10].

β-tricalcium phosphate (bTCP) is an absorbable ceramic that has been widely used for bone reconstruction due to its bioactive and osteoconductive properties [11–13]. On the other hand, type I collagen (Col I) is inherently biocompatible and biodegradable and promotes cellular adhesion and proliferation [14]. For these properties Col I has been extensively tested for both bone and cartilage repair [2, 15].

In most studies, scaffolds have been used in combination with cells from different sources. Mesenchymal stromal cells (MSCs) have become attractive for cartilage and bone tissue engineering [15] because of their easy isolation, expansion, self-renewal ability and multipotential differentiation properties [16, 17]. Therapies based on MSCs or MSC-derived products to treat human diseases have yet to be tested in large animal models before starting clinical trials [18].

Preclinical studies in orthopaedic research using sheep as a large animal model are becoming common [2, 18–24]. This is due to the marked similarities of the sheep with human bone/cartilage regeneration processes, joint structure, and weight bearing; thus ovine large animal models have potential in translational research [24, 25].

Knowledge of ovine MSCs is recently increasing and the sheep genome sequence was recently completed [26], aiding in obtaining understanding of these cells. However, characterization of ovine MSCs is not well established [19] and controversy exists among the results [27].

To date, most cartilage/bone engineering studies developed in both animal and human models have shown heterogeneous results [28] and the analyses have usually focused at the neotissue level, not at the cellular level.

In this work we performed an ovine bone marrow MSC (oBMSCs) characterization using surface marker expression and multipotent differentiation. The osteogenesis of oBMSCs cultured on bTCP and Col I scaffolds was tested in depth by histological and ultrastructural analyses. Moreover, the chondrogenic repair capacity of oBMSCs cultured on Col I scaffolds was evaluated using an *in vitro* cartilage repair model.

## Materials and Methods

### Ovine sample procurement

This study was approved in accordance with the Ethics Committee for Animal Experimentation of the CHUAC. For all experiments mixed breed of domestic sheep (*Ovis aries*) at an age of 1 to 6 years were used (males and females from 5150760107901, Lamas, A Coruña, Spain) (n = 7, 5 females and 2 males). The animals were housed in the veterinary care facility under standardized conditions of humidity and temperature with a 12 h light/dark rhythm. Animals included in this research were slaughtered in accordance with the guidelines of the Experimental Surgery Unit of the Training Technology Centre from Complexo Hospitalario Universitario de A Coruña (CHUAC), Spain.

Bone marrow samples used to obtain oBMSCs were isolated from iliac crest aspirates of sheep (n = 6). Samples of femoral condyles were obtained from previously slaughtered sheep (n = 3).

### Isolation and culture of ovine bone marrow stromal cells

Iliac crest bone marrow aspirates were collected into centrifuge tubes (*Costar Corning Incorporated*, *New York*, *USA*) and centrifuged after clotting with culture medium (Dulbecco's Modified Eagle's Medium; DMEM; *Lonza*, *Madrid*, *Spain*) supplemented with 5% fetal bovine serum (FBS; *Gibco- Thermo Fisher Scientific*, *Madrid*, *Spain*) and 1% penicillin/streptomycin (P/S; *Gibco*) (5%FBS/DMEM). The supernatants were discarded. Under sterile conditions, the marrow clots were divided with tweezers into several parts on adherent culture plates (*Costar Corning Incorporated*) and grown in culture medium 20%FBS/DMEM at 37°C in a humidified atmosphere with 5% CO_2_.

After 48 hours, the plates were washed with sterile saline solution (*Fresenius Kabi*, *Barcelona*, *Spain*), eliminating non-adherent cells and clots, and culture medium was added. The culture medium was then replaced every 3 days.

When cell confluence reached 80%, subculturing was performed for cell expansion. A 15-min preplating technique [29] was performed at the first cell passage to eliminate any remaining macrophages or fibroblasts from the culture.

At the 2^nd^-8^th^ passages, cells were used for morphological, phenotypical and functional culture studies.

### Phenotypic characterization using flow cytometry

At passages 3^rd^, 4^th^, and 8^th^ after culture expansion, cells from 3 animals were analyzed by flow cytometry. A total of 2x10^5^ cells were transferred to fluorescence-activated cell sorting (FACS) polypropylene tubes (NUNC^TM^, *VWR International*, *Radnor*, *Pennsylvania*). The antibodies used for these experiments are listed in Table 1. The antibodies used are specific for markers associated with mesenchymal and hematopoietic lineages. A minimum of 1x10^5^ cell events per assay was acquired using a FACsCalibur flow cytometer (*BD Biosciences*, *Madrid*, *Spain*). Data were analyzed using Cell Quest software (*BD Biosciences*) and the results are expressed as mean of positive percentage ± standard error.

**Table 1:** Antibodies used for flow cytometry.

ISOTYPES			
Antibody	Clone	Specificity	Source
FITC Mouse IgG1Kappa Isotype Control	MOPC-21		BD Pharmingen^TM^
PE Mouse IgG1 Kappa Isotype Control	MOPC-21		DB Pharmingen^TM^
PECy5 Mouse IgG1 Isotype Control	1F8		Abcam
Rabbit anti-mouse Inmunoglobulins/FITC			DAKO
**LABELED PRIMARY ANTIBODIES**			
PE Mouse Anti-Human CD34 monoclonal	581	Hematopoietic progenitor cell antigen 1 (HPCA1)	BD Pharmingen^TM^
Mouse Anti-Sheep CD44:FITC monoclonal	25.32	Homing cellular adhesion molecule (HCAM)	AbD Serotec
PE Anti-Human CD29-PE monoclonal	TS2/16	β1 Integrin	BioLegend
FITC Mouse Anti-Rat CD45 monoclonal	OX-33	Leukocyte common antigen (LCA)	BD Pharmingen^TM^
FITC Mouse Anti-Human CD45 monoclonal	HI30	LCA	BD Pharmingen^TM^
PE Rat Anti-Mouse CD45 monoclonal	30-F11	LCA	BD Pharmingen^TM^
Purified Mouse Anti-Human CD69 monoclonal	FN50	Very early activation antigen	BD Pharmingen^TM^
PE Mouse Anti-Human CD73 monoclonal	AD2	Esto-5´-nucleotidase	BD Pharmingen^TM^
PE-Cy^TM^5 Mouse Anti-Human CD90 monoclonal	5E10	Thy-1 membrane glycoprotein	BD Pharmingen^TM^
Mouse Anti-Human CD105:FITC monoclonal	SN6	Endoglin, SH2	AbD Serotec
PE-Cy^TM^5 Mouse Anti-Human CD106 monoclonal	51-10C9	Vascular cell adhesión molecule 1 (VCAM-1)	BD Pharmingen^TM^
PE Mouse Anti-Human CD166-PE monoclonal	3A6	Activated leukocyte cell adhesion molecule (ALCAM)	Immunostep
**NON-LABELED PRIMARY ANTIBODIES**			
Mouse Anti-Human CD45 monoclonal	MEM-28	LCA	Abcam
Anti-CD90	MRC OX-7	Thy1	Abcam
Rabbit anti-CD90 polyclonal		Thy1	Antibodies-online.com
Mouse Anti-Human CD105	105C02	SH2	Abcam
Mouse Anti-Human CD271 monoclonal	ME20.4	Anti-Nerve Growth Factor Receptor (NGFR p75)	Sigma
Anti-Human/Mouse SSEA-4 monoclonal	MC-813-70	Stage-specific embryonic antigen 4 (SSEA-4)	R&D Sytems
Mouse Anti-Human STRO-1	NS1-Ag4-1	Stromal antigen 1 (STRO-1)	*Developmental Studies Hybridoma Bank*
**SECONDARY ANTIBODIES**			
Rabbit Anti-Mouse Immunoglobulins/FITC			DAKO
Goat anti-rabbit IgG-PE			Santa Cruz Biotechnology, INC.

Antibodies used for phenotypical characterization by flow cytometry. FITC = Fluorescein isothiocyanate; PE = Phycoerythrin.

### Multipotential characterization of ovine bone marrow stromal cells

At 2^nd^-5^th^ passages, oBMSCs from 3 animals were differentiated towards the three mesenchymal lineages: adipocyte, osteoblast and chondrocyte.

#### Adipogenic and osteogenic differentiation

oBMSCs were seeded at 5x10^3^ cells/cm^2^ in a chamber slide (*BD Falcon*^*TM*^, *BD Biosciences*) for histology (n = 4) and at 50x10^3^ in a 6-well-plate (*Costar Corning Incorporated*) for molecular analyses (3 independent samples). Adipogenesis was induced by culturing for 21 days in human MSC (hMSC) Commercial Adipogenic Differentiation Medium (*Lonza*), following the manufacturer’s instructions, with 1ng/ml of Rosiglitazone (*Sigma Aldrich Quimica SA*) added. Osteogenesis was induced by culture for 21 days in hMSC Commercial Osteogenic Differentiation Medium (*Lonza)*, following the manufacturer’s instructions.

Both adipogenic and osteogenic differentiations were compared to a control consisting of cells cultured for the same period of time in 20% FBS/DMEM. Differentiations were confirmed by staining techniques and gene expression quantification using Real Time-PCR (qPCR).

#### Chondrogenic differentiation

Chondrogenesis was assessed using the micropellet formation (2.5x10^5^ cells) technique [30]. oBMSCs (from 3 independent samples) pellet was cultured in hMSC Commercial Chondrogenic Differentiation Medium (*Lonza*) with 10 ng/ml of human transforming growth factor (TGFβ-3) (*Prospec-Tany Technogene Ltd*., *Rehovot*, *Israel*) for 21 days, following the manufacturer’s instructions. Chondrogenic differentiation was compared with cell micropellets cultured for the same period of time with 20% FBS/DMEM. After 21 days, two cell aggregates were *OCT* (*Tissue-Tek cryo-OCT compound*, *Thermo Fisher Scientific*) embedded and frozen for histology and three cell micropellets were frozen, without OCT, for molecular analyses.

### Bone and cartilage engineering

Two different types of scaffolds were used: horse tendon Col I sponges and bTCP synthetic ceramic. Col I sponges (6 mm diameter x 1 mm thick) were supplied by the Italian company, *Opocrin*, *SPA*, *Corlo di Formigine-Modena*. They were used to test both osteogenic differentiation and chondrogenic repair capacities.

1000–2000 μm grain bTCP (*Macrobone*, *Euroteknika Iberia*, *Barcelona*, *Spain*) was provided by the Instituto Coruñés de Implantología y Rehabilitación Oral (ICIRO) and used to test osteogenic differentiation.

### Osteogenic differentiation on Col I sponges and on beta-tricalcium phosphate ceramic

oBMSCs from 3 animals at the 3^rd^ passage were cultured on the surface of sponges (200,000 cells/cm^2^) (2 replicates) and oBMSCs (2x10^5^) from 3 animals at the 4^th^ passage were also cultured with 100 mg of grain 1000–2000 μm bTCP ceramic (2 replicates), both for 30 days. Osteogenic differentiation was induced by culturing the oBMSCs in hMSC Commercial Osteogenic Differentiation Medium, following the manufacturer’s instructions. In addition, each type of scaffold, without cells, was cultured in the same medium and for the same period of time to serve as a negative control.

#### Cartilage lesion repair using Col I sponges

Ovine cartilage was obtained from femoral condyles (from 3 animals) and excised with a 6 mm biopsy punch (7 discs; *Kai Medical*, *Solingen*, *Germany*). Lesions (3 mm) were made with a dental driller (*Gebr*. *Brasseler Gmbh & Co*. *KQ*, *Lemgo*, *Germany*) with a rotor (*EWL K9*).

oBMSCs (2x10^5^) from 2 animals (4^th^ passage) were seeded on Col I sponges for 30 min at 37°C, and the construct was then placed inside the lesion. Punches were cultured for 8 weeks using hMSC Chondrogenic Differentiation Medium with 10 ng/ml of human TGFβ-3.

### Molecular analysis of cell differentiation

Differentiation of oBMSCs towards the three mesenchymal lineages (adipocyte, osteoblast and chondrocyte) was analyzed using molecular biology techniques.

Isolation of total RNA from cell culture was accomplished using Trizol Reagent (*Invitrogen*^*TM*^*—Thermo Fisher Scientific*), following the manufacturer’s protocol. RNA was assessed for quantity at 260 nm using a NanoDrop^TM^ spectrophotometer (*Thermo Fisher Scientific*).

The RT-PCR reaction was performed from total RNA following the manufacturer’s instructions, using the SuperScript^TM^ First-Strand Synthesis System for RT-PCR (*Invitrogen* ^*TM*^) in a Thermocycler (*Gene Amp PCR System 9700*, *Applied Biosystems—Thermo Fisher Scientific*).

qRT-PCR analyses were performed, using the primers and conditions shown in Table 2, on a LightCycler^®^ 480 Instrument (*Roche*, *Basel*, *Switzerland*) using LightCycler 480 SYBR Green I Master (*Roche*), following manufacturer’s instructions.

**Table 2:** Primers used for the relative expression (REL) of typical genes for differentiation and multipotency.

Gene name	Primer Sequences	Product size	Reference Sequence
GAPDH	5’ ATCCTGCCAACATCAAGTGG 3’	84 nt	NM_001190390.1
	5’ CAGCCTTCTCCATGGTAGTGA 3’		
VIM	5’ ACATCGAGATCGCCACCTAC 3’	100 nt	KC904793.1
	5’ TTGGTTTCCCTCAGGTTCAG 3’		
SOX2	5’ CGAGGGAATGGACCTTGTATAG 3’	88 nt	X96997.1
	5’ CTGCAAAGCTCCTACCGTATC 3’		
LPL	5’ TGAAACTTGGCAAAGCTACAGA 3’	78 nt	DQ016298.1
	5’ GGCGTCTTTTGTAAAAGTTACCTCAT 3’		
FABP4	5’ GGATGTGGTCAACATTAAATCAGA 3’	95 nt	EU301804.1
	5’ TGTCATCTGGAGTGACTTCATCA 3’		
OP	5’ GCAGTCCTCACTGTCACAAGA 3’	105 nt	AF152416.1
	5’ TGCTGTGGAATTAGCAGTCG 3’		
OCN	5’ GAAGAGACTCAGGCGCTACCT 3’	107 nt	DQ418490.1
	5’ CATCACAGTCAGGGTTGAGC 3’		
AGG	5’ TTTGGACTTTGGCAGAATACC 3’	78 nt	FJ200438.1
	5’ AATCCAGAAGGAAGACCACTTG 3’		
COL II	5’ GGGCGAGACTGTGATTGAGT 3’	118 nt	FJ378650.1
	5’ GACAGGCCCTATGTCCACAC 3’		
COL I	5’ CCTGGATGCCATTAAGGTCT 3’	113 nt	AF129287.1
	5’ TCTTGTCCTTGCTCTTGCTG 3’		

Product size in nucleotides (nt) and GenBank reference. Housekeeping gene: Glyceraldehyde-3-phosphate Dehydrogenase (GAPDH). To evaluate grade of multipotency: Vimentin (VIM) and SRY (sex-determining region Y)-box 2 (SOX2). For adipogenesis: Lipoprotein Lipase (LPL) and Fatty Acid Binding Protein 4 (FABP4). For osteogenesis: secreted Phosphoprotein 1 (OP) and Osteocalcin (OCN). For chondrogenesis: Aggrecan (AGG) and type II Col alpha 1 (COL II). For fibroblasts: type I Col alpha 1(COL I).

Data analysis was performed for triplicates using the LightCycler 480 Relative Quantification software (*Roche*) and REL were calculated by the 2^-ΔΔCt^ method [31].

### Cytological and histological analyses

#### Assessment of adipogenic, osteogenic and chondrogenic differentiation of oBMSCs

For adipogenesis evaluation, differentiation was confirmed by detection of cytoplasmic lipid droplets by Oil red O (OR-O) staining. For osteogenesis evaluation, differentiation was analyzed by Alizarin red (AR) staining, to assess the presence of calcium deposits.

For chondrogenesis evaluation, cell aggregates were stained with hematoxylin and eosin (HE), Masson's trichrome (MT), PAS-Alcian blue (PAS-AB) and Safranin O (SO) for Col and proteoglycans (PG). Moreover, inmunostaining for Col I, Col II and aggrecan (Agg) (Table 3) was performed. The peroxidase/DAB ChemMateTM DAKO EnVision^TM^ detection kit (*Dako*, *Barcelona*, *Spain*) was used.

**Table 3:** Antibodies used for immunohistochemistry.

Antibody	Clone	Specificity	Source
Anti-Collagen I monoclonal	COL-I	Type I Collagen	Abcam
Anti-Collagen II monoclonal	5B2.5	Type II Collagen	Abcam
Anti-Aggrecan ARGxx monoclonal	BC-3	Aggrecan	Abcam
Anti-Osteocalcin monoclonal	OC4-30	Osteocalcin	Abcam

Antibodies used for the analysis of chondrogenic and osteogenic differentiation by immunohistochemistry.

#### Assessment of osteogenic constructs and chondrogenic lesion repair

Assessment of osteogenic constructs and chondrogenic lesion repair was performed by histology and electron microscopy analyses.

Osteogenic cell and cell-free scaffolds, and chondrogenic repair models were fixed in 4% formaldehyde and embedded in paraffin.

To evaluate osteogenic constructs, samples were decalcified and stained with HE, Von Kossa (VK) and AR. Immunostaining for Col I and osteocalcin (OCN) was also performed.

For cartilage repair evaluation, samples were stained as described for “Chondrogenesis evaluation”. For assessment of the quality of ovine cartilage repair, the histology scoring system, ICRS II, which was designed for use in human cartilage repair evaluation, was used. Some modifications to this grading system were made for adaptation for the analysis of ovine cartilage repair. Our modified score system comprised 7 of the 14 ICRS II parameters [32].

Quantitative analyses of the staining and immunostaining described above were measured using ImageJ 1.48v (*National Institutes of Health*, *Bethesda*, *USA*). After the colour substraction of non-stained and counterstained regions, the percentage of stained area was measured and expressed as mean ± standard error. Also, NIS-elements AR 3.0 software (Nikon, Netherlands) was used for AR quantification.

Samples for electron microscopy were processed in the *Servizo de Apoio á Investigación* at the University of A Coruña (SAI-UDC). For scanning electron microscopy (SEM), scaffolds were first fixed in 3% glutaraldehyde in cacodylate buffer. Before further fixation in osmium tetroxide, the scaffolds were dehydrated in a graded series of ethanol. Samples were critical-point dried by flooding with liquid CO_2_ in a Bal-Tec CPD 030 (BAL-TEC, *Balzers*, Liechtenstein) and gold-sputtered with the coater Bal-Tec SCD 004 (BAL-TEC). Samples were observed using a scanning electron microscope, JSM 6400 (*JEOL*, *Tokyo*, *Japan*). For transmission electron microscopy (TEM) scaffolds were first fixed in 3% glutaraldehyde in cacodylate buffer and dehydrated in a graded series of acetone and then fixed in osmium tetroxide. Samples were embedded in Spurr (*Electron Microscopy Sciences*, *Hatfield*, *USA*) and 2 μm sections were stained with toluidine blue to confirm the presence of cells. Then, 60-nm-thick Spurr sections were made and observed using a transmission electron microscope, Jeol JEM 1010 (*JEOL*). For Energy Dispersive X-ray analysis (EDX), 5-μm-thick Spurr sections were obtained and deposited on a slide for SEM. Samples were coated with a thin layer of pure graphite carbon using BAL-TEC CEA 035 equipment (*BAL-TEC*). Samples were subsequently analyzed using a scanning electron microscope (*JEOL JSM-6400*, *JEOL*) equipped with a chemical microanalysis system through energy dispersive x-ray *OXFORD INCA ENERGY 200 (Oxford instruments*, *Abingdon*, *England*).

### Statistical analyses

All statistical analyses were performed with GraphPad Prism (*GraphPad Software*, *Inc*.), using non-parametric tests (Mann-Whitney *U* and Kruskal- Wallis tests). *p-values* <0.05 are considered to be statistically significant. Results are expressed as the mean ± standard error.

## Results

### Morphologic characterization of oBMSCs

**Fig 1:**
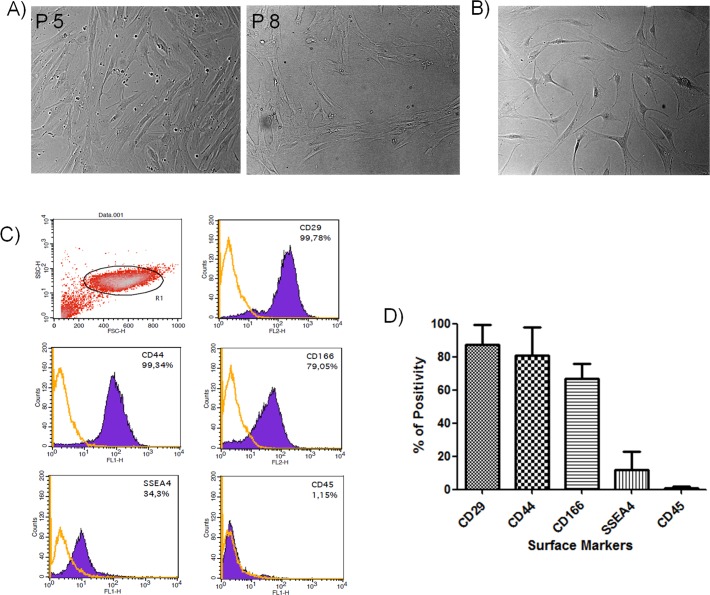
Morphologic and phenotypic characterization of oBMSCs. (A) Image of ovine bone marrow mesenchymal stromal cells at passage 5 and 8 isolated from iliac crest aspirates. (B) Image of human bone marrow mesenchymal stromal cells (hBMSCs) at passage 10 isolated from bone marrow aspirates, as previously described [33]. Both images show the typical spindle-shaped fibroblast-like morphology characteristic of the MSC. Original magnification x100. (C) Phenotypic characterization by flow cytometry of a representative population of oBMSCs, for markers characteristic of MSCs and hematopoietic cells that react with sheep. The purple line signifies the specific antibody, while the orange line represents the isotype control. Plots correspond to a representative experiment (D) Bar graph representing the percentage average values (mean ± standard error) of positivity of the markers analyzed from 3 samples at different passages (at 3^rd^, 4^th^ and 8^th^ passages); markers characteristic of MSCs (CD29, CD44, CD166), embryonic cells (SSEA-4), and hematopoietic cells (CD45).

oBMSCs were successfully isolated from sheep bone marrow by puncture of the iliac crest. It took 14±2 days for first passage cells and this cell population showed the typical spindle-shaped fibroblast-like morphology characteristic of the MSC, with irregular cytoplasm and numerous cytoplasmic prolongations (Fig 1A). The isolated oBMSCs also showed adherence to plastic in culture and were very similar to those from human sources (Fig 1B)[33].

### Immunophenotypic characterization of oBMSCs

Immunophenotypic analysis was performed to assess the expression of mesenchymal and hematopoietic markers in isolated oBMSCs. This analysis was performed on cells from 3 samples at different passages (passage 3 through 8). The cell-surface antigen profile of oBMSCs was analyzed using antibodies directed against human (CD29, CD34, CD45, CD69, CD73, CD90, CD105, CD106, CD166, CD271, SSEA4 and STRO1) and rat (CD45 and CD90) CD surface markers because of the restricted number of antibodies are available for sheep (only anti-sheep CD44 was used). Although oBMSCs have cell surface antigens similar to hBMSCs, most antibodies reacting with hBMSCs did not show reactivity with oBMSCs (S1 File), despite of checking several antibody clones (CD45, CD90 and CD105, S1A Fig). Three exceptions were found (Fig 1C): CD29, which showed 87.58%±11.70% positivity; CD166, which showed 66.85%±8.79% positivity; and SSEA4, which showed 11.67%±11.31% positivity. Anti-sheep CD44 showed 81.08%±16.68% positivity (S1 File). oBMSCs have shown to be negative for CD45 (even when positive in ovine blood, S1B Fig), indicating no contamination with cells of hematopoietic origin (Fig 1C).

### *In vitro* differentiation potential of oBMSCs

#### Adipogenic differentiation of oBMSCs

**Fig 2:**
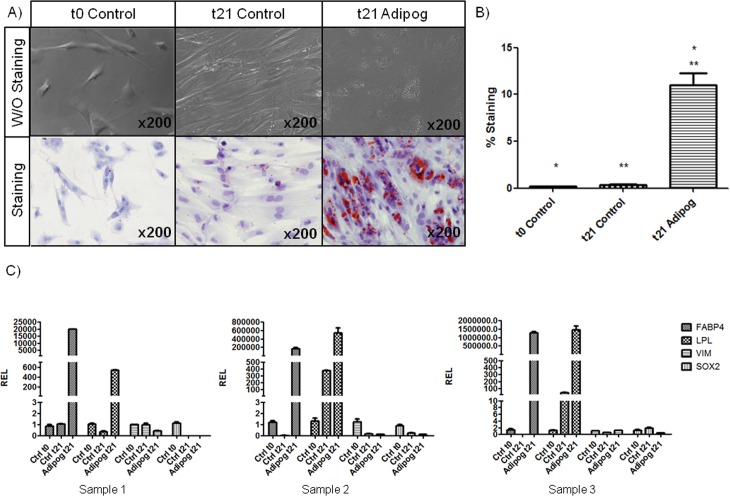
Adipogenic differentiation of oBMSCs. (A) Ovine bone marrow mesenchymal stromal cells (oBMSCs, n = 4, passages 2^nd^-5^th^) at day 0 (left) of differentiation and cultured for 21 days in non-differentiation control medium (20% FBS/DMEM; middle) and adipogenic-differentiation medium (right). The presence of adipocytes was assessed by detection of lipid drops using Oil Red O (OR-O) stain. Magnification x200. (B) Bar graph represents the percentage of area positively stained for OR-O expressed as the mean ± standard error. * and ** indicates p<0.05. (C). mRNA levels were measured in three independent samples (n = 3) by quantitative Real Time Polymerase Chain Reaction (qRT-PCR), as described in Materials and Methods. Data are expressed as mean ± standard error of the relative expression (REL). The results are normalized to values obtained for oBMSCs at day 0 of differentiation, considered to equal 1.

Adipogenic differentiation was assessed by OR-O staining at 0 and 21 days of culture in adipogenic medium (Fig 2A and 2B). Stimulated oBMSCs showed positive staining with single adipocytic multivacuolar cells secreting lipid droplets. However, 0 and 21 day- non-stimulated oBMSCs, showed the absence or much weaker staining for lipids than stimulated oBMSCs (*p* = 0.001). Adipogenic differentiation was also assessed by qRT-PCR (S1 File). The expression levels of the adipogenic lineage-specific genes, LPL and FABP4, were studied. The expression levels of two multipotent-specific genes, VIM and SOX2 (Fig 2C), were also assessed. oBMSCs stimulated for 21 days in adipogenic medium showed an increased relative expression (REL) of the genes FABP4 and LPL in all the three samples, compared with oBMSCs at day 0 of differentiation. Considering the expression of the multipotency genes in the stimulated oBMSCs at 21 days, the expression levels of VIM and SOX2 decreased compared with oBMSCs at day 0 day of differentiation in two out of three samples (Sample 2 and 3, Fig 2C). Only one sample (Sample 1, Fig 2C) of oBMSCs cultured for 21 days in control medium (20% FBS/DMEM) showed a decrease in the expression of FABP4 and LPL, compared with oBMSCs at day 0 of differentiation. At 21 days in two out of three oBMSCs (Sample 1 and 2, Fig 2C) cultured in control medium, we found that the expression levels of VIM and SOX2 were decreased compared with oBMSCs at day 0 of differentiation.

#### Osteogenic differentiation of oBMSCs

**Fig 3:**
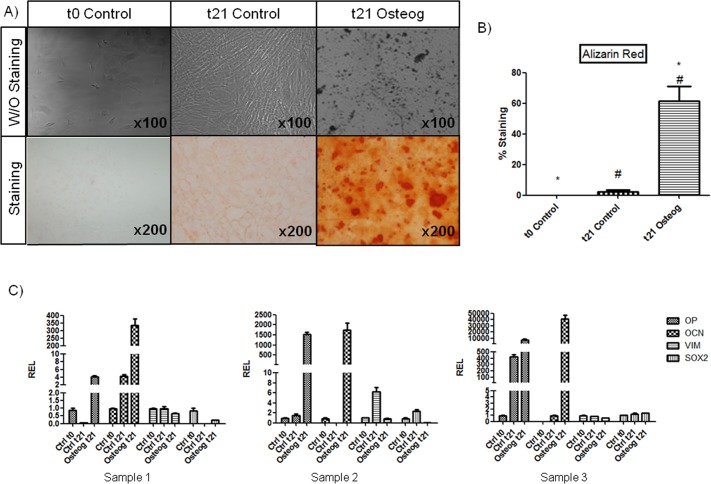
Osteogenic differentiation of oBMSCs. (A) Ovine bone marrow mesenchymal stromal cells (oBMSCs, n = 4, passages 2^nd^-5^th^) at day 0 of differentiation (left) and after culture for 21 days in non-differentiation control medium (20% FBS/DMEM, middle) and osteogenic-differentiation medium (right) (Magnification x100). The presence of osteoblasts was assessed by detection of calcium deposits using Alizarin Red (AR) stain (Magnification x200). (B) Bar graph represents the percentage of area positively stained for AR expressed as the mean ± standard error. 1. * and # indicates p<0.05. (C) The osteogenic differentiation potential was confirmed by quantitative Real Time Polymerase Chain Reaction (qRT-PCR). The expressions of osteogenic- and multipotency-specific genes were analyzed. mRNA levels were measured in three independent samples (n = 3) by qRT-PCR as described in Materials and Methods. Data are expressed as mean ± standard error of the relative expression (REL). The results are normalized to values obtained for oBMSCs at day 0 of differentiation, considered to equal 1.

The osteogenic differentiation potential of oBMSCs was examined by determining the presence of calcification using AR stain (Fig 3A and 3B). 21-day-stimulated oBMSC populations showed extracellular calcium deposition. Non-stimulated oBMSCs, maintained in control medium (20% FBS/DMEM) for 0 and 21 days, showed less positivity (*p* = 0.000) for AR staining.

Osteogenic differentiation was also assessed by qRT-PCR (S1 File). The expression level of the osteogenic lineage-specific genes OP and OCN were studied. In addition, the expression levels of multipotency-specific genes VIM and SOX2 (Fig 3C) were analyzed. oBMSCs stimulated for 21 days in osteogenic medium showed an increased expression of OP and OCN in all the three samples compared with oBMSCs at day 0 of differentiation. The expression levels of SOX2 and VIM at 21 days were lower in two out of three oBMSCs stimulated samples (Sample 1 and 2, Fig 3C) than at 0 day of differentiation. oBMSCs cultured for 21 days in control medium also showed an increased expression of OP compared with oBMSCs at day 0 of differentiation, except in sample 1 (Fig 3C). In samples 1 and 3 (Fig 3C) the expression of VIM at 21 days in 20% FBS/DMEM was approximately the same as that of oBMSCs at day 0 of differentiation. SOX2 gene expression in 21 days control medium compared to day 0 was variable among all samples (Fig 3C).

#### Chondrogenic differentiation of oBMSCs

**Fig 4:**
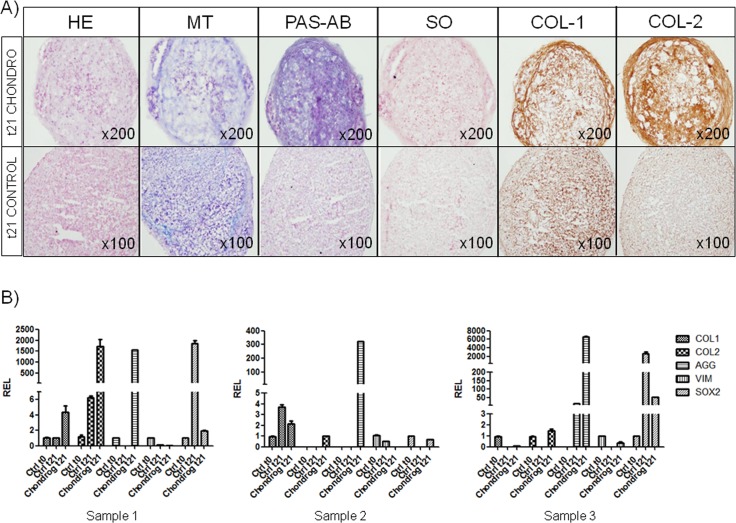
Chondrogenic differentiation of oBMSCs. (A) Ovine bone marrow mesenchymal stromal cells (oBMSCs, n = 3, passages 2^nd^-5^th^) in micromass culture for 21 days in non-differentiation control medium (“t21 Control”; 20% FBS/DMEM) and chondrogenic-differentiation medium (“t21 Chondro”) (Magnification x100 and x200, respectively). Micromasses were stained with hematoxylin-eosin (HE), Masson´s trichrome (MT), PAS-Alcian blue (PAS-AB) and safranin O (SO). Immunodetection of type I and type II collagen (Col I and Col II) was assessed on micromasses. (B) The chondrogenic differentiation potential was confirmed by quantitative Real Time Polymerase Chain Reaction (qRT-PCR). The expressions of chondrogenic- and multipotency-specific genes were analyzed. Data are expressed as mean ± standard error of the relative expression (REL). The results are normalized to values obtained for oBMSCs at day 0 of differentiation, considered to equal 1.

Indicating chondrogenic differentiation, HE staining revealed the rounded morphology of the micromass cultures. Stimulated micropellets showed higher cellularity in the core than that found in non-stimulated cultures. However, both possessed extracellular matrix (ECM), although total Col (Fig 4A, MT) and PG (Fig 4A, PAS-AB and SO) were more abundant in stimulated cultures. Immunostaining for Col I and II was also more prominent in the stimulated micropellets (Fig 4A, Col I and Col II).

Chondrogenic differentiation was also assessed by qRT-PCR (S1 File). The expressions of the chondrogenic lineage-specific genes COL II and AGG were tested. To eliminate differentiation towards fibroblasts, COL I was tested as well. Furthermore, the expression levels of two multipotency-specific genes, VIM and SOX2 (Fig 4B), were also analyzed. oBMSCs stimulated for 21 days in chondrogenic medium showed an increased expression of the genes AGG, COL I and COL II, compared with oBMSCs at day 0 of differentiation in all the samples, excepting sample 3, in which COL I decreased (Fig 4B). At 21 days, the expression level of SOX2 was slightly increased in samples 1 and 3, whereas the expression level of VIM was decreased in all the samples, compared with oBMSCs at day 0 of differentiation (Fig 4B). oBMSCs from samples 2 and 3 cultured for 21 days in control medium did not show expression of COL II, whereas oBMSCs from sample 1 showed slightly positivity compared with day 0. On the other hand, expression of AGG was not detected either in oBMSCs from samples 1 and 2, meanwhile little positivity was found in sample 3. Furthermore, COL I expression changed compared among the samples. Measuring the expression of the multipotency genes in the oBMSCs cultured in control medium for 21 days, the expression of VIM was decreased compared with oBMSCs at 0 day of differentiation. Additionally, SOX 2 expression was significantly increased in oBMSCs from samples 1 and 3 cultured for 21 days in control medium (Fig 4B).

### *In vitro* osteogenic differentiation of oBMSCs cultured on collagen sponges and β-tricalcium phosphate ceramic

#### Histological and immunohistochemical analyses of the osteogenic constructs

**Fig 5:**
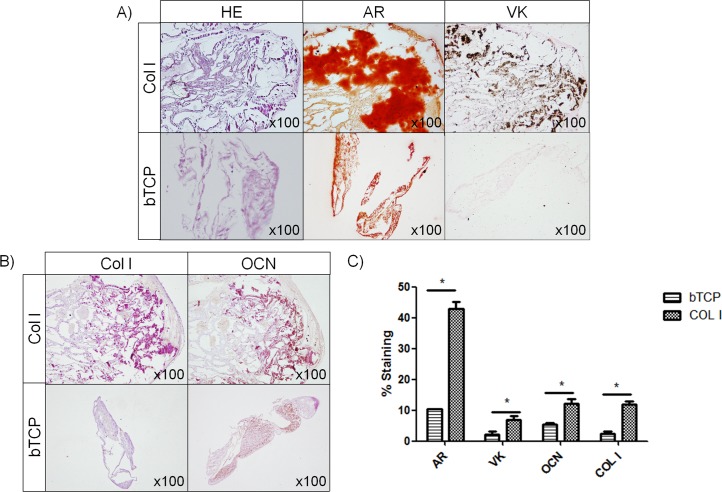
Osteogenic differentiation of ovine bone marrow mesenchymal stromal cells (oBMSCs) cultured on Col I sponges and β-tricalcium phosphate (bTCP) ceramic. (A) Histological evaluation of osteogenic constructs (oBMSCs at passages 3^rd^ and 4^th^, n = 3) stained with hematoxylin-eosin (HE), Alizarin Red (AR) and Von Kossa (VK). Magnification x100. (B) Immunohistochemical analysis of the osteogenic constructs immunostained for type I collagen (Col I) and osteocalcin (OCN). Magnification x100. (C) Bar graph represents the percentage of cells positive for AR, VK staining and OCN and Col I immunostaining, expressed as the mean ± standard error. * indicates p<0.05

Osteogenic constructs showed low cellularity on both types of scaffolds, with scant ECM formation. However, Col I sponges had cells with a cubic morphology resembling osteoblasts (Fig 5A and 5B). Staining for AR and VK was significantly higher on Col I sponges than on bTCP ceramics (42.99%±2.25% and 10.30%±0.24%; *p-value* = 0.012, respectively for AR and 6.97%±1.31% and 2.21%±0.93%; *p-value* = 0.024, respectively for VK) (Fig 5A and 5C). Immunostaining for Col I and OCN revealed significantly higher positivity for both proteins on Col I sponges than on bTCP scaffolds (11.85%±1.07% and 2.47%±0.61%; *p-value* = 0.002, respectively, for Col I protein and 12.22%±1.46% and 5.51%±0.51%; *p-value* = 0.002, respectively, for OCN protein) (Fig 5B and 5C).

#### Ultrastructural analysis of osteogenic constructs

**Fig 6:**
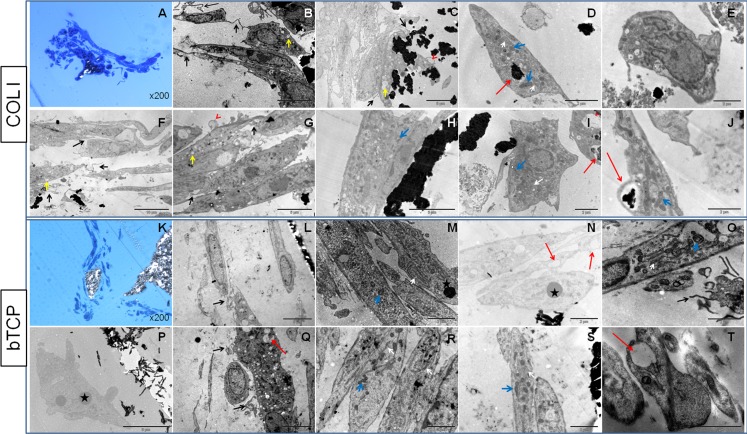
Transmission electron microscopy. Images obtained from type I collagen (Col I) and β-tricalcium phosphate (bTCP) ceramic constructs (oBMSCs at passages 3^rd^ and 4^th^, n = 2). The different scaffolds are shown in rows. The first two rows (from A to J) are Col I sponges and the last two rows (from K to T) are bTCP constructs. A, K: semi-thin sections (1 μm) of the constructs embedded in Spurr and stained with toluidine blue (Magnification x200). Scales of bars: F, 10 μm; B, C, G, H, L, P, Q, 5 μm; D, E, I, J, M, N, O, R, S, 2 μm; T, 1 μm. Black arrows: prolongations; yellow arrows: vacuoles; red arrows: precipitates inside vacuoles; red arrowheads: empty vacuoles; white arrows: rough endoplasmic reticulum; blue arrows: mitochondria; black stars: electron-dense spheres.

Constructs of Col I sponges, analyzed by TEM, showed cells (Fig 6A in dark blue) surrounding the calcium phosphate precipitates (Fig 6A in black and white). Cells exhibited differing morphologies; round- (Fig 6E), oval- (Fig 6B), star- (Fig 6I) and elongated-shaped (Fig 6D, 6H and 6J), and remained as aggregates (Fig 6B, 6C, 6F and 6G). In these aggregates, cells, containing few small prolongations (black arrows), were observed to be in contact with each other. Increased numbers of vacuoles (yellow arrows) existed at the contact areas between cell membranes. Many calcium phosphate precipitates were observed, not only in the cytoplasm of the cells (Fig 6D and 6J), but also in the extracellular space (Fig 6B, 6C, 6F, 6H and 6I). In the cytoplasm, and before being secreted outside the cell, these precipitates appeared inside vacuoles (Fig 6D, 6J and 6I; red arrows). Occasionally, these vacuoles were empty, perhaps because the precipitate had already been secreted outside the cell (Fig 6C, 6I and 6G; red arrowheads). In general, cells were organelle-rich. Granular endoplasmic reticula with ribosomes (Fig 6D and 6I; white arrow), mitochondria with cristae (Fig 6D, 6J, 6I and 6H; blue arrows) and euchromatic nuclei (Fig 6C, 6E, 6G and 6I) were observed. In addition, in the cytoplasm of differentiated cells, the accumulation of multiple matrix vesicles was observed (Fig 6C).

The ultrastructural analysis of bTCP ceramic showed cells (Fig 6K in dark blue), with an elongated-shape (Fig 6L, 6M, 6R and 6S) and small prolongations (Fig 6L, 6O and 6Q; black arrows) surrounding the calcium phosphate precipitates (Fig 6K in black and white). These cells formed aggregates (Fig 6M and 6R) and showed accumulations of roughly fused spherical material in the cytoplasm (Fig 6M, 6N and 6P; black star). These accumulations were electron-dense because of their high osmium tetroxide affinity, indicating high lipid content. Furthermore, these accumulations were more abundant on bTCP ceramic than on Col I sponges. Calcium phosphate deposits were only observed outside the cell (Fig 6L, 6N, 6P and 6S), although it was not possible to distinguish between deposits secreted by the differentiated cells and precipitates from the bTCP ceramic. In addition, in the cytoplasm of the cells, multiple empty vacuoles that could have initially contained such deposits were observed (Fig 6N, 6T and 6Q; red arrows). In general, cells showed an organelle-rich composition (Fig 6M, 6O, 6Q, 6R and 6S) with multiple vesicles (Fig 6L, 6M, 6N and 6Q) and euchromatic nuclei (Fig 6L, 6O and 6Q).

#### Energy dispersive X-ray study of Col I constructs

**Fig 7:**
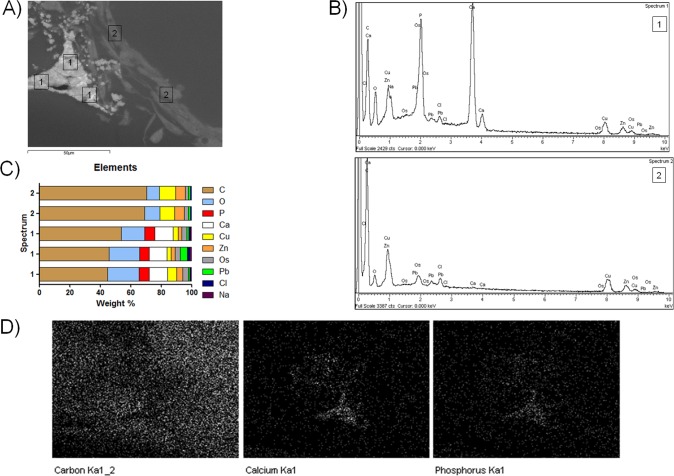
Energy dispersive X-ray (EDX) analysis performed on the type I collagen (Col I) constructs. (A) Images obtained by scanning electron microscopy (SEM) showing the two areas analyzed; the number “1” indicates a precipitate, and the number “2” indicates a cell aggregate. (B) Spectrum of the two regions analyzed showing the elements detected: Spectrum 1 corresponding with a precipitate and spectrum 2 with a cell aggregate. (C) Bar graph represents the percentage of the elements found in five different analyzed spectra; three from the analyzed precipitate and the other two from the cell aggregate. (D) Map of C, Ca and P distribution analyzed in the whole sample.

To verify that the deposits found in the extracellular space and in the cytoplasm of the cells cultured on Col I sponges were composed of calcium phosphate, an energy dispersive X-ray (EDX) study was performed. The analysis was performed at different areas of the sample preparations to ascertain its exact composition. These areas included a region of the precipitate (Fig 7A, highlighted with the number “1”) and a region of cells (Fig 7A, highlighted with the number “2”). As can be seen in Fig 7B, 7C and 7D, the main elements found in the precipitate were carbon (47.95%±4.93%) and oxygen (18.77%±2.86%) followed by calcium (11.87%±0.41%) and phosphate (6.51%±0.19%). These elements were, however, not detected in the region of cells (Fig 7B, 7C and 7D); instead, carbon (69.725%±0.95%) and oxygen (9.08%±1.02%) followed by copper (10.11%±0.56%) and zinc (6.41%±0.11%) were found. The EDX analysis was not performed on the bTCP ceramic constructs because it was not possible to distinguish between the deposits secreted by the differentiated cells and the precipitates from the bTCP ceramic.

#### Morphometric analysis of osteogenic constructs

**Fig 8:**
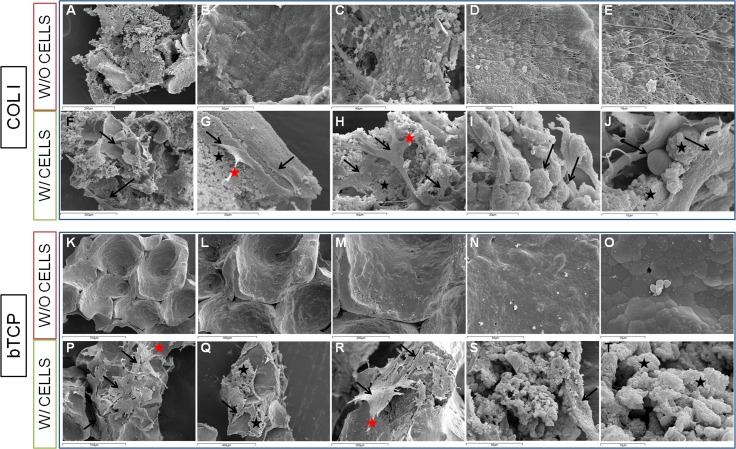
Scanning electron microscopy (SEM). SEM images obtained from type I collagen (Col I) and β-tricalcium phosphate (bTCP) ceramic constructs (oBMSCs at passages 3^rd^ and 4^th^, n = 2). Cell-free Col I sponges (W/O CELLS, A-E) and Col I constructs with cells (W/CELLS, F-J) are shown in the two first rows. Cell-free bTCP scaffolds (W/O CELLS, K-O) and bTCP constructs with cells (W/ CELLS, P-T) are shown in the last two rows. Scales of bars: K, P 700 μm; L, Q 400 μm; A, F, M, R 200 μm; B, G, N, S 50 μm; C, H 40 μm; D, I 20 μm; E, J, O, T 10 μm. Black arrows: cells; black stars: extracellular matrix; red stars: cell prolongations.

Cell-free Col I sponges (Fig 8A–8E) showed by SEM that their general structure were fibrous whereas bTCP (Fig 8K–8O) ceramics were smoother. Because of the lack of microporosity, cells grew on and adhered to the surface of bTCP, but not inside, whereas the Col I structure had allowed cell growth throughout the scaffold, as observed in different sections. On Col I constructs, cells showed different morphologies from flattened (Fig 8F, 8G and 8H; black arrows) to rounded (Fig 8I and 8J; black arrows), but only flattened cells were seen on bTCP (Fig 8P–8S; black arrows). Cells presented small prolongations on both scaffolds (Fig 8G, 8H, 8P and 8R; red stars).

Furthermore, more volume of ECM was observed in Col I constructs (Fig 8G–8J; black stars), compared with bTCP constructs (Fig 8Q, 8S and 8T; black stars).

### Chondrogenic differentiation of MSCs cultured on collagen sponges in an *in vitro* articular cartilage repair model

**Fig 9:**
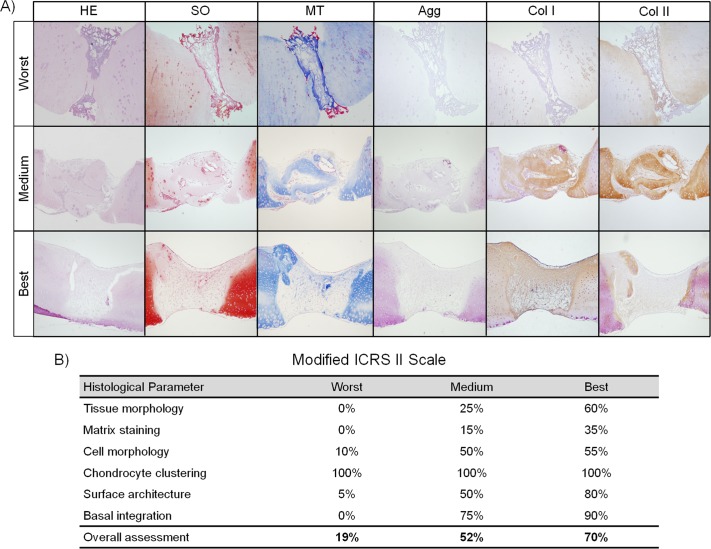
Chondrogenic differentiation of ovine bone marrow mesenchymal stromal cells (oBMSCs) on type I Col (Col I) sponges in an *in vitro* cartilage repair model. (A) Images of the histological analyses performed in the best, intermediate and worst replicas (oBMSCs at 4^th^ passage, n = 2; 7 cartilage discs from 3 animals), stained with hematoxylin-eosin (HE), Safranin O (SO), Masson´s trichrome (MT) and immunostained for aggrecan (Agg), collagen type I and II (Col I and Col II). Inmunostainings were counterstaining with H-E. Magnification x40. (B) Table that represents the cartilage repair scoring according to the modified ICRS II scale [32] of each replica.

Although our first goal in the articular cartilage repair model was to produce semicircular lesions on the cartilage surface, deeper lesions (from side to side) were generated in some of the replicas (Fig 9A) because of the thinness of the ovine cartilage. Several replicas also had slivers of native cartilage inside the lesion (Fig 9A; medium results, second row) because of drill movement during handling. Three representative replicas of worst, intermediate and best neotissue were evaluated by the ICRS II scale from a total of 7 (2 best, 2 intermediate and 3 worst) (Fig 9A and 9B).

HE staining allowed the observation of neotissue formation inside the lesion (Fig 9A, intermediate and best replicas). Col I scaffolds ranged from barely- (worst replica) to nearly totally- degraded (best replica) (Fig 9A).

The first parameter analyzed in the adapted ICRS II scoring system, using MT staining, was “tissue morphology” (Fig 9). This criterion was assessed by analyzing the collagen fiber distribution. The worst results did not show any ECM, scoring a value of 0%. Intermediate results showed heterogenic and obvious collagen fibers, being an intermediate tissue between fibrocartilage and fibrous tissue (25% score). In the best results, collagen fibers were more evident in the deep zone of the repaired tissue than in the superficial zone (60% value) (Fig 9A and 9B).

The second criteria used to evaluate PG content was “matrix staining” (Fig 9), which was assessed using SO staining. The results ranged from 0% (the worst) to 35% (the best).

The next two variables, analyzed by HE staining, were “cell morphology” and “chondrocyte clustering” (S2A Fig). In the worst results, 10% of the cells were rounded, while in the intermediate and best results, approximately 50–55% of the cells were rounded. All the replicas showed absence of clusters, scoring 100%.

The next item studied was “surface architecture.” The worst results showed disruptions on the surface (5%); while intermediate results revealed an upper surface smoother than the lower one (50%). The best replicas showed lack of disruptions, delaminations and loosenings, scoring 80%. The final criterion analyzed, “integration with native cartilage,” ranged from 0% (worst results) to 75–90% (intermediate and best replicas, respectively). Finally, to score “overall assessment,” the statistical mean of the values for all 6 parameters analyzed was calculated. The worst cartilage repair score was 19%, indicating fibrous tissue; the intermediate cartilage repair scored 52%, indicating a fibrocartilage tissue; and finally, the best neotissue formed was graded in the midrange of fibrocartilage and hyaline cartilage (70%).

Some replicas have been immunostained for Col I, Col II and Agg. The collagen content of the repaired tissue was absent in the worst results, whereas in the intermediate and best replicas, both ECM proteins were present (Fig 9A). Aggrecan was also absent in the worst results, but slightly present in the other two results.

## Discussion

Previous studies have reported the isolation of oMSCs from bone marrow [5, 23–25], adipose tissue [34–36], umbilical cord blood [36, 37], peripheral blood [21, 38], amniotic fluid [39, 40], dermis [41] and periodontal ligament [42]. Despite the increasing use of sheep as a large animal model for tissue engineering, because of their similarities to humans in size, joint architecture and healing mechanisms [25], ovine MSCs have been poorly characterized compared to human MSCs [24]. Accordingly, the aim of this study was to first perform an extensive morphologic, immunophenotypical and functional characterization of isolated oBMSCs. Second, the *in vitro* osteogenic differentiation of oBMSCs cultured on Col sponges and bTCP ceramic was studied, and finally, the chondrogenic differentiation of oBMSCs cultured on Col sponges in an *in vitro* articular cartilage repair model was analyzed.

For our study, oBMSCs were obtained from iliac crest aspirates, the most common approach described in the literature, to collect bone marrow [2, 13, 15, 17, 23–25, 43]. For oBMSCs isolation, the simplest and easiest protocol for successful isolation of hBMSCs was used, omitting two steps sometimes applied in other oBMSCs isolation protocols: density gradient centrifugation [2, 18, 19, 25, 34, 44–46] and erythrocyte lysis [15]. Several reports simply assumed that cells isolated from bone marrow were oBMSCs, omitting any further extensive characterization process [2].

In our study, cells with adherence to culture plastic and a typical spindle-shaped fibroblast-like morphology were isolated. As Rentsch *et al*. [46], Al Faqeh *et al*. [17] and McCarty *et al*. [25] described, we also observed that these cells presented prolongations and typical fibroblast-like morphology. oBMSCs have been reported to be smaller [46] and to show an *in vitro* higher proliferation rate and longer life span [28] than their human counterparts, although we did not test these parameters.

Following the International Society of Cell Therapy criteria [47], hMSCs have to express at least the CD73, CD90 and CD105 cell surface markers. There is little information about the cell surface antigen profile of isolated oBMSCs because of the limited availability of antibodies specific for sheep [25]. This lack of information is also due to human antibodies not having cross-reactivity with sheep antigens. Therefore, when using human antibodies, the absence of expression of such cell surface markers needs to be interpreted with caution [27]. The antigen expression pattern of oBMSCs has been reported to be positive for anti-human CD44 and CD105, and negative for anti-ovine CD45 antibodies [5]. Adamzyk *et al*. [19] observed only in some specimens that the CD73 expressed by the oBMSCs which was able to bind the selected anti-human CD73 antibody, and Martínez-Lorenzo *et al*. [35] observed high positivity for anti-human CD90, but very low positivity for anti-human CD73 and CD105 antibodies. In agreement with the results published in the literature, antibodies with human reactivity for CD73 [18], CD90 [18, 25] and CD105 [18, 25, 44] did not react positively with our oBMSCs. This absence of expression may be due to the lack of cross reactivity of these antibodies between species. Consistent with the literature, we found absence of anti-rat CD45 positivity [5, 25, 44], indicating that the cells were not of hematopoietic origin. Moreover, we observed uniformly high cross-reactivity for antibodies anti-human CD29, anti-ovine CD44 and anti-human CD166, as described by others [13, 23, 25, 44]. These markers have been associated with human bone marrow stromal, adipose and dental pulp cells [23]. oBMSCs isolated by Zannettino *et al*. [28] also were positive for antibodies anti-ovine CD29 and anti-mouse CD44. Furthermore, we found positivity for an embryonic stem cell marker, hSSEA4, but it was not expressed consistently in all the samples. Therefore, taking these results together, the oBMSCs cell-surface antigen profile seems similar to the expression profile of hBMSCs, in that they express markers of mesenchymal and embryonic stem cells [33, 48].

In the literature, there are some studies that did not perform any functional characterization of oBMSCs [2, 15], isolating these cells only by culture-plastic adherence. Other studies assessed differentiation potential into only one cell lineage, including Martínez-Lorenzo *et al*. [35], who just tested chondrogenic differentiation, and Reichert *et al*. [23], who just tested osteogenic differentiation. Some studies have assessed the capacity of oBMSCs to differentiate into the three lineages [16, 28, 44–46], while other studies have performed both histological and RT-PCR analysis of differentiation [5, 25]. In other reports, published before the complete sequencing of the sheep genome [26], multiple sequence alignment of bovine, human, and rat, among other species, were used for RT-PCR primer design [2, 20, 25]. Therefore we performed a multipotent differentiation analysis in which qRT-PCR primers were synthesized, based only on the sheep genome, to quantify specific genes for each cell lineage. Uniquely in our study, assessments of the gene expression of multipotency markers, such as VIM and SOX2, were also assessed. These studies were performed at 0 and 21 days of differentiation, not only in stimulated differentiation media, but also in non-stimulated control DMEM medium.

To achieve adipogenesis, a human differentiation medium was initially used without success (data not shown), confirming results obtained by Mrugala *et al*. [5]. Their report indicated that the stimulus needed to induce adipogenic differentiation differs for human and ovine cells. Thus, adipogenic differentiation did not occur when oBMSCs were cultured in the media typically used to induce hBMSC adipogenesis. Complementation of the differentiation media with dexamethasone and rosiglitazone was needed to trigger the ovine adipogenic differentiation process [5, 16, 49]. In our experimental culture conditions, including dexamethasone and rosiglitazone, oBMSCs differentiated adipogenically, as shown by our observations of cytoplasmic lipid droplets, an trended to increase expression of adipogenic genes (LPL and FABP4), and a to decrease expression of multipotency (VIM and SOX2) genes. Our results are in accordance with previous reports [5, 21, 24, 25, 28, 44, 45]. The effect of adipogenic culture media on differentiation of oBMSCs was studied by Adamzyk *et al*. [19]. These authors obtained oBMSC adipogenesis without donor variations, but with a larger variation due to media used. In contrast, they found that variation in osteogenic differentiation was influenced by donor, protocol and preculture-dependent variations. After 21 days of osteogenic stimulation, we histologically observed the presence of calcification and an increased expression of OP and OCN at the gene level. These results agree with previous reports [5, 16, 21, 23–25, 28, 44]. Moreover, after 21 days in control culture media, we also observed slight AR staining and increased expression of the OP and OCN specific-lineage genes in some samples. In the absence of stimulation, high basal levels of OP were also obtained by Mrugala *et al*. [5], possibly due to growth factors that stimulate osteogenesis in the FBS included in their culture media [50].

We observed that oBMSCs were able to differentiate towards chondrocyte-like cells only when TGFβ3 was added to the chondrogenic culture medium, as previously reported by Mrugala *et al*. [5]. However, Martínez-Lorenzo *et al*. [35] did not obtain good chondrogenesis using TGFβ3 exclusively, finding it necessary to add bone morphogenetic protein-6 (BMP-6). In our study, oBMSCs were highly positive for CD44, a putative marker of enhanced chondrogenic capacity [51]. The ability of oBMSCs to trigger chondrogenesis was assessed using a pellet culture system, as previously reported [5, 15, 24]. This three-dimensional culture enables stronger cell-to-cell contact [52] allowing increased presence of Col II throughout the matrix [16]. Histologically, we found the presence of PG and Col in the ECM of pellet cultures. The expression of AGG, one of the main PGs of ovine [20] and human [53] cartilage ECM was only evident in stimulated cells, indicating the success of oBMSC chondrogenesis.

Applications of MSCs, especially in combination with biomaterials, have raised optimism for future therapies to repair tissues including cartilage and bone [19]. TCP resorbable ceramics are widely used as bone fillers in orthopaedic surgeries for treatment of long bone fractures [28]. Gupta *et al*. [54] compared cell-enriched TCP grafts, TCP alone, and autografts in an *in vivo* model. They found that TCP alone could not serve as an effective graft in the absence of osteoprogenitor cells, while autologous bone marrow with TCP increased bone formation. El-Jawhari *et al*. [55] found that the addition of Col to natural bovine bone scaffolds improved the attachment, survival and proliferation of hBMSCs. Using histology and electron microscopy, we observed more cell attachment, ECM deposition and osteogenic phenotype in Col sponges than in bTCP ceramics. Although our cells express high levels of CD29 we found poor distribution of cells within Col I scaffolds. Some subclasses of this marker are associated with more efficient binding to Col-based scaffolds [28]. The use of Col I scaffolds possesses various beneficial properties for clinical application, including biodegradability, low immunoreactivity and enabling the transport of nutrients [15, 56]; these properties have led to their wide use in tissue engineering [56].

Histologically, we found osteoblasts in Col constructs, as previously described in *in vivo* bone repair using MSCs with platelet-rich plasma (PRP)[34]. We also confirmed the presence of osteoblasts in Col constructs by detecting OCN, a bone protein exclusively synthesized by these cells [57]. Moreover, VK and AR staining confirmed the presence of bone cells by the specific presence of insoluble calcium phosphate salts [57] and calcium deposits [58] in the extracellular spaces.

The EDX analysis provides a useful tool for elemental quantification in tissues, allowing the semi-quantification of the amounts of Ca and P in bone [59]. In our study, we performed an EDX analysis to confirm the presence of phosphate-calcic precipitates secreted by osteogenic differentiated cells in Col constructs. We obtained the same Ca/P ratio in these deposits as that described by Perdikouri *et al*. [59] in a rat fracture healing model. Melrose *et al*. [60] also reported that Ca and P were the major elements detected in calcic deposits formed in sheep intervertebral discs.

Scanning electron microscopy showed different cell morphologies on bTCP and Col biomaterials. Schmitt *et al*. [57] also found different morphologies depending on the surface topography of different types of scaffolds. Thus, in some biomaterials cells were capable of growth inside the scaffold while in others, cells were flattened and more dispersed on the surface. On bTCP, oBMSCs were also flattened but dispersed along the outer side of the biomaterial, whereas in Col biomaterial, cells could grow throughout the scaffold because of its higher porosity. Using TEM, we observed that osteogenic differentiated oBMSCs acquired an osteoblast-like phenotype on both biomaterials, as also described by Ozen *et al*. [61]. Desantis *et al*. [62] found two cell types in oBMSC pellets, electron-dense and electron-lucent, while we only observed one cell type. Our osteogenic differentiated cells were similar to their electron-lucent counterparts.

*In vitro* testing provides standardized and quantifiable information about cytotoxicity, cell proliferation and differentiation capacity rate more easily than *in vivo* testing. Moreover, it is accepted that *in vitro* testing is used as a first stage test to avoid the unnecessary use of animals in materials testing [63]. However, it is impossible to create *in vitro* bone repair models because it is not possible to maintain long-term bone cultures.

Several authors [15, 20] have developed chondrogenic *in vitro* studies using ovine cells and different types of scaffolds. Endres *et al*. [20] cultured ovine chondrocytes on polyglycolic acid (PGA)-fibrin biomaterials. They found that chondrocytes were surrounded by ECM, rich in PG, and had the homogenous distribution of Col II characteristic of hyaline cartilage. Schulz *et al*. [15] tested the *in vitro* formation of cartilaginous grafts using Col I hydrogels and ovine cells. They compared the phenotype of constructs from ovine chondrocytes and oBMSCs and achieved better results using the latter. However, these previous studies did not test the constructs in a native cartilage environment as we did. We created an ovine cartilage repair model and evaluated the capacity of repair using oBMSCs and Col I constructs.

Using a modified ICRS II scale [32], our construct replicas obtained scores from 19% (fibrous tissue) to 70% (fibrocartilage/hyaline cartilage) out of 100% (hyaline cartilage). We should point out that this scale was designed to evaluate *in vivo* osteochondral human repair. On one hand, only histological parameters were taken into account. On the other hand, in human cartilage the presence of chondrocyte clusters is associated with a pathological state, unlike ovine cartilage where it is usual to find more than one chondrocyte per lacuna (S2B Fig) [20, 32].

In our work, variability between replicas was observed. The small lesion size due to the thinness of ovine cartilage could contribute to this variability between replicas [2]. It is necessary to consider that the generation of the lesion disrupts cartilage ECM and that can cause lost of PGs and collagens, as we could observe by immunostaining. Other limitations of this study may be the lost of PGs because of the culture [64], time of culture and the absence of mechanical stimuli.

We used shorter culture times (2 months) than those described in the literature [65]. Mathematical models have predicted a timeframe *in vivo* of at least 18 months to obtain completely mature cartilage. This agrees with clinical studies that demonstrate that cartilage repair is a slow process [65]. However, longer culture times considerably increase the risk of culture contamination and cartilage degradation [66]. Likewise, the absence of mechanical stimuli may impact adversely on the quality of newly formed repair tissue. However, our model allows analyzing cartilage samples from the same donor using different study variables, and testing engineered constructs in the native cartilage environment.

In conclusion, the results from the present study demonstrated that oMSCs have been effectively isolated from bone marrow aspirates and showed morphological, phenotypical and functional properties similar to those observed in their human counterparts. Further optimization would be needed to use Col I scaffolds as temporary matrices for cell proliferation, migration, and osteochondral differentiation of oBMSCs. Additionally, in an *in vitro* cartilage repair model, we demonstrated that oBMSCs cultured on Col I sponges can successfully form fibrocartilage/hyaline cartilage tissue. Finally, this study suggests that oBMSCs may have potential use in osteochondral engineering.

## Supporting Information

S1 Fig Phenotypic characterization of oBMSCs. (A) Phenotypic characterization by flow cytometry of a representative population of oBMSCs for markers characteristic of MSCs and hematopoietic cells, that did not show positivity and/or reactivity with oBMSCs (B) Phenotypic characterization by flow cytometry of mononuclear cells from ovine blood, for different clones of anti-CD45 antibody.
(TIF)

S2 Fig Histology of the cartilage and *in vitro* cartilage model. A) Images of hematoxylin-eosin (H-E) staining and type II Collagen (Col II) immunostaining performed in the best, intermediate and worst replicas. B) Images of H-E, Masson’s Thricrome (MT) and Safranin O (SO) staining and Col II, Col I and Aggrecan (Agg) immunostaining, performed in a cultured biopsy of ovine cartilage.
(TIF)

S1 File Individual data points underlying means and deviations of: (Sheet “Cytometry”) flow cytometry percentage of positivity results, (Sheet “qRT-PCR”) Relative expression (REL) of different genes for multipotent differentiation analyzed by real time PCR and, (Sheet “%Staining”) histological and immunohistochemical percentage of area stained or immunostained. (XLSX)
