# Supplementary figures and images for: Ovine Mesenchymal Stromal Cells: Morphologic, Phenotypic and Functional Characterization for Osteochondral Tissue Engineering
Source: PLoS One. 2017 Jan 31;12(1):e0171231. doi: 10.1371/journal.pone.0171231 (PMC5283731; doi:10.1371/journal.pone.0171231)

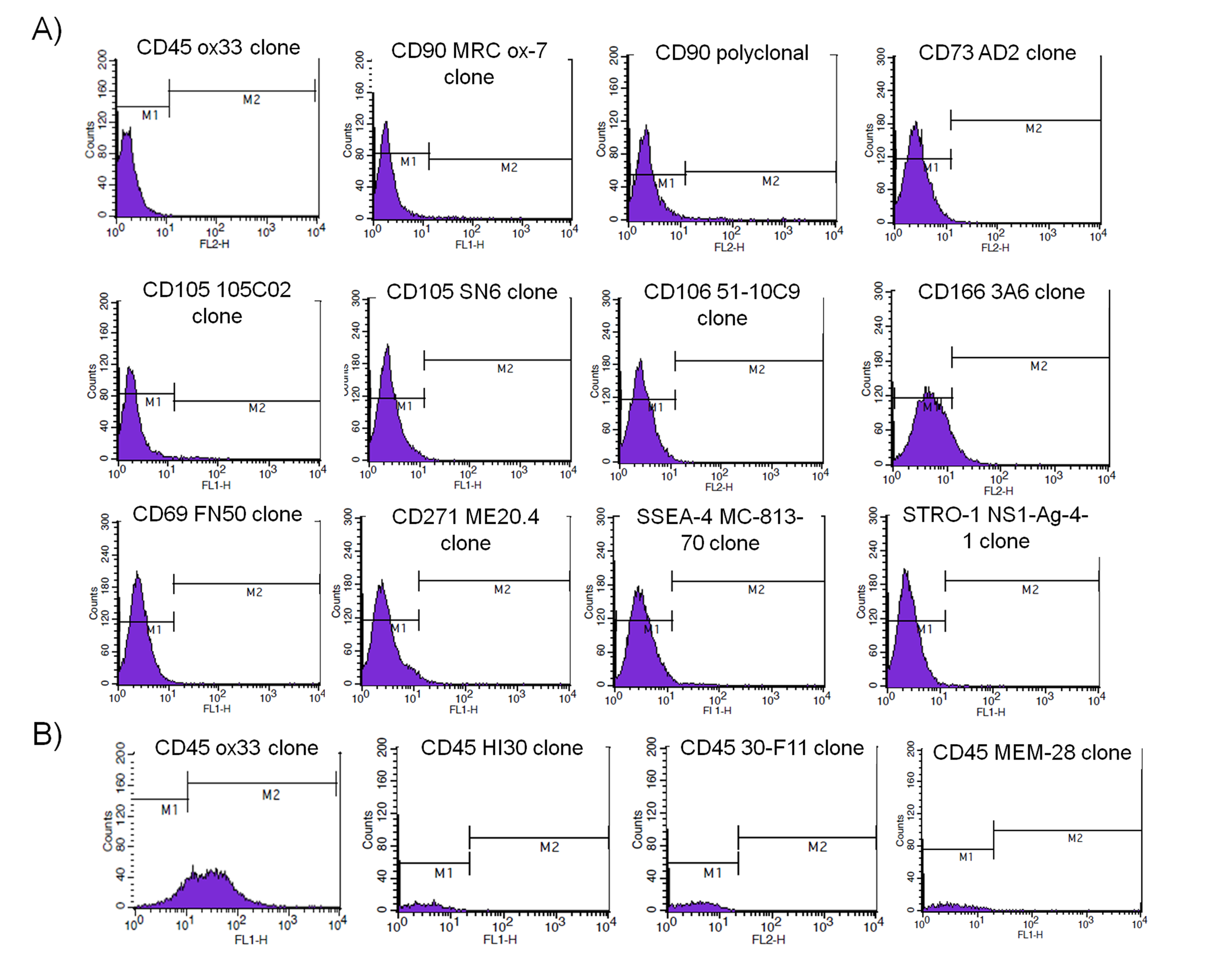

Supplement: S1 Fig — (A) Phenotypic characterization by flow cytometry of a representative population of oBMSCs for markers characteristic of MSCs and hematopoietic cells, that did not show positivity and/or reactivity with oBMSCs (B) Phenotypic characterization by flow cytometry of mononuclear cells from ovine blood, for different clones of anti-CD45 antibody. (TIF) [file pone.0171231.s001.tif]

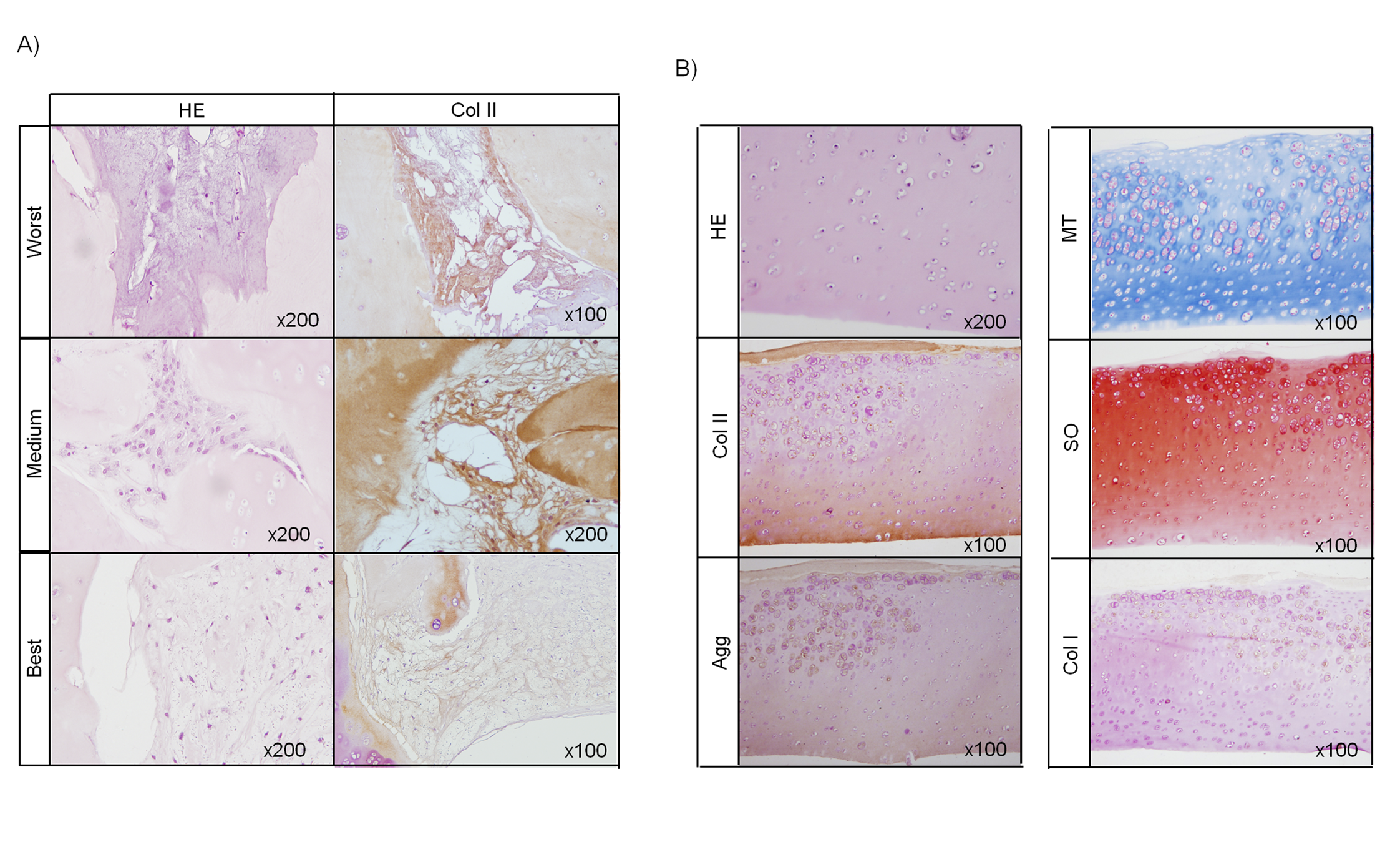

Supplement: S2 Fig — A) Images of hematoxylin-eosin (H-E) staining and type II Collagen (Col II) immunostaining performed in the best, intermediate and worst replicas. B) Images of H-E, Masson’s Thricrome (MT) and Safranin O (SO) staining and Col II, Col I and Aggrecan (Agg) immunostaining, performed in a cultured biopsy of ovine cartilage. (TIF) [file pone.0171231.s002.tif]
